# Thrombosis in the portal venous system caused by hypereosinophilic syndrome: A case report

**DOI:** 10.1097/MD.0000000000013425

**Published:** 2018-11-30

**Authors:** Jinfeng Lin, Xiaoying Huang, Weihua Zhou, Suyan Zhang, Weiwei Sun, Yadong Wang, Ke Ren, Lijun Tian, Junxian Xu, Zhilong Cao, Zunguo Pu, Xudong Han

**Affiliations:** a Department of Critical Care Medicine, Nantong Third People's Hospital, Nantong University; b Department of Critical Care Medicine, Hai’an County People's Hospital, Nantong, China.

**Keywords:** anticoagulation, extensive thrombosis in the portal venous system, gastrointestinal bleeding, hypereosinophilic syndrome, thrombolysis

## Abstract

**Rationale:**

Extensive thrombosis in the portal venous system caused by hypereosinophilic syndrome (HES) is rare, and there is no consensus on anticoagulant and thrombolytic treatments for arteriovenous thrombosis caused by HES.

**Patient concerns:**

The clinical data of a patient with extensive thrombosis in his portal venous system (superior mesenteric, splenic, hepatic, and portal veins), renal artery thrombosis, and mesenteric thrombosis caused by HES with secondary gastrointestinal bleeding and intestinal necrosis were retrospectively analyzed. Before admission, his eosinophil count increased to 7.47 × 10^9^/L, and HES had been confirmed via bone marrow cytology. The patient experienced fever, cough, abdominal pain, massive hematemesis, and hematochezia that developed in succession. Abdominal computed tomography showed portal vein and superior mesenteric vein thromboses.

**Diagnosis:**

Hypereosinophilic syndrome; extensive thrombosis in the portal venous system; acute eosinophil-associated pneumonia; gastrointestinal bleeding; intestinal necrosis.

**Interventions:**

The patient was first treated with methylprednisolone, plasma exchange/hemofiltration, and single or combined use of unfractionated heparin and argatroban for anticoagulation. He was also administered alteplase and urokinase, successively, for thrombolytic treatment. Once the thromboses finally disappeared, the patient underwent surgery to excise a necrotic intestinal canal.

**Outcomes:**

The thromboses disappeared with these treatments, and the patient recovered after the necrotic intestinal canal was excised.

**Lessons:**

The clinical manifestations of HES are complex and varied, and this condition can cause severe and extensive arteriovenous thrombosis. Anticoagulation therapy and thrombolysis are necessary interventions, and appear to be safe and effective.

## 1 Introduction

Hypereosinophilic syndrome (HES) is a group of conditions characterized by increased peripheral blood eosinophils (>1.5 × 10^9^/L) and lesions infiltrating the tissues and organs. HES is rare, with an incidence of approximately 0.036 to 6.3 per 100,000 population.^[1]^ Eosinophil infiltration can lead to multiple organ injuries and results in diverse clinical manifestations.^[2]^ The heart, respiratory system, skin, gastrointestinal tract, and nervous system are commonly affected; clinical manifestations include heart failure, pneumonia, skin deformities, and neurological impairment.^[3]^ The incidence of extensive deep venous thrombosis caused by HES is approximately 25%, and the associated mortality rate is 5% to 10%. Thrombosis can occur in locations such as the heart, lung, abdominal cavity, brain, skin, and deep veins of the lower extremities.^[4]^ Although the incidence of deep venous thrombosis caused by HES is low, the prognosis is often poor when such thrombosis is extensive. To date, there have been no reports of HES patients with concurrent extensive thrombosis in the portal venous system and renal arteries, and no consensus has been reached on the most appropriate anticoagulant and thrombolytic treatments of arteriovenous thrombosis caused by HES. Herein, we report a critically ill patient with extensive thrombosis in the portal venous system and renal arteries caused by idiopathic HES; he also experienced concurrent gastrointestinal bleeding and intestinal necrosis. The patient was cured by successive administration of unfractionated heparin and argatroban for anticoagulant treatment as well as alteplase and urokinase for thrombolytic treatment. This patient's disease course can provide a case study reference in the diagnosis and treatment of HES.

## 2 Case presentation

**Figure 1:**
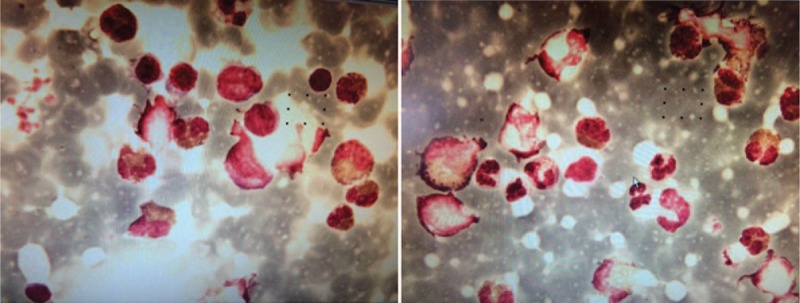
Bone marrow cytological examination. Granulocyte proliferation was markedly high, and the proportion of eosinophils was significantly increased. The morphologies of the remaining granulocytes at various stages were normal, with erythroid hyperplasia inhibited slightly.

The patient was a 22-year-old unmarried man who worked in construction. He had lived in a basement for a long time before developing his symptoms. The patient began experiencing coughing and expectoration that did not resolve through self-medication; he thus visited Xuzhou Central Hospital on June 29, 2017. Chest computed tomography (CT) showed inflammation in both lungs. The patient then visited the Hai’an County People's Hospital, where he was diagnosed with lung infection and was prescribed moxifloxacin. No significant improvements in cough and expectoration were observed, and the patient developed a fever (his highest temperature was 39.3 °C) along with bloody sputum, and his eosinophil count continuously increased to a maximum of 7.47 × 10^9^/L. Cytological examination of his bone marrow indicated HES (Fig. 1); no fusion gene detection was performed, and examinations of extractable nuclear antibody series, vasculitis, nephropathy-related antibodies, immunoglobulins, and complement showed no abnormal results. After ruling out allergic pneumonia and vasculitis-related pneumonia caused by drugs and parasites, the patient was deemed to have acute eosinophil-associated pneumonia, and was thus treated with additional methylprednisolone starting on July 14, 2017. His coughing and expectoration improved while his body temperature decreased. On July 17, the patient experienced abdominal distension and periumbilical pain with persistent colic that progressively worsened; he also had nausea and vomiting of the contents of the stomach. Physical examination indicated periumbilical tenderness as well as rebound tenderness. Plain abdominal radiography in an upright position showed no obvious abnormalities, and the patient was treated with acid suppression, spasmolysis, and induced defecation. The patient also had elevated D-dimer, and mesenteric arteriovenous thrombosis could not be ruled out; hence, he was administered low molecular weight heparin for anticoagulant treatment, but no significant improvements of his abdominal symptoms were observed. On July 19, the patient experienced palpitations, nausea, hematemesis, and hematochezia combined with decreased blood pressure and a heart rate of 179 beats/min (the latter were evidence of hemorrhagic shock caused by gastrointestinal bleeding). The patient was treated with blood transfusion, fluid infusion, and norepinephrine to maintain his blood pressure. Blood examinations showed that fibrinogen levels decreased to 0.18 g/L and D-dimer levels were 40 mg/L; abdominal CT indicated thrombosis in the portal and superior mesenteric veins.

**Figure 2:**
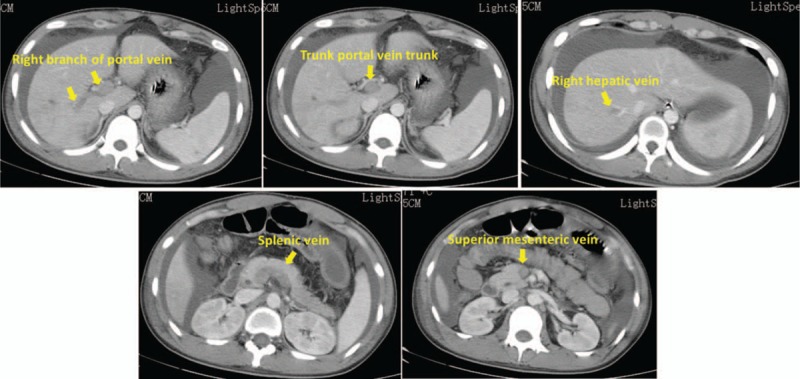
Whole abdominal enhanced computed tomography scan on July 21, 2017. The arrow in the figure refers to veins with extensive venous filling defects in the contrast-enhanced scan during the venous phase, suggesting venous thrombosis.

As the patient was now in critical condition, he was admitted to our hospital for treatment on July 19. The patient's test indicators before his admittance are shown in Table 1. He had previously been healthy, and had no history of diseases such as allergic rhinitis, urticaria, or bronchial asthma nor of allergies to drugs or foods. He also had no family history of thrombotic diseases. Upon admission, he had a body temperature of 37.4 °C, heartrate of 140 beats/min, respiratory rate of 38 breaths/min, blood pressure of 130/80 mmHg (with norepinephrine 10 μg/min, dopamine 7 μg/kg min, vasopressin 2 U/h), and peripheral capillary oxygen saturation of 96%. The superficial lymph nodes were normal, and there was no obvious skin rash anywhere on his body. However, he had an obvious abdominal bulge, and the abdomen itself was soft with periumbilical tenderness, no rebound pain, positive mobility dullness, and weak bowel sounds. On post-admission examination, the patient's tumor markers were negative; moreover, all rheumatoid factors as well as anti-nuclear, anti-histone, anti-double strand DNA, anti-nucleosomal, anti-Sjogren syndrome, anti-scleroderma, anti-phospholipid, and anti-neutrophil cytoplasmic antibodies were negative. A fecal test (performed at another hospital) was negative for parasites and eggs. The results of a coagulation test were as follows: prothrombin time, 63.0 seconds; international normalized ratio, 5.48; activated partial thromboplastin time, 303.70 seconds; antithrombin 3, 69.8%; fibrin degradation products, 137.3 mg/L; and D-dimer, 67.55 mg/L. Enhanced abdominal CT showed multiple emboli in the portal, right hepatic, splenic, and superior mesenteric veins. Furthermore, a portion of the small intestine was enlarged, and the intestinal wall was thickened due to edema (Fig. 2). Chest CT revealed low perfusion zones in pulmonary tissue and bilateral atelectasis of the lower lobes with possible pulmonary infarction. His preliminary diagnoses at admission was HES; thrombosis in the portal, superior mesenteric, right hepatic, and splenic veins; gastrointestinal bleeding; and hemorrhagic shock.

**Table 1:**
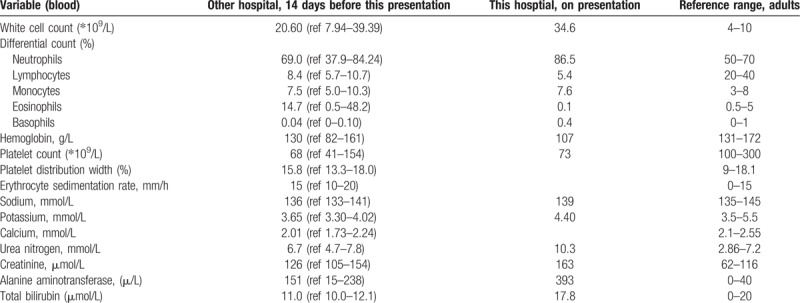
Test indicators of the patient before and at admission.

**Figure 3:**
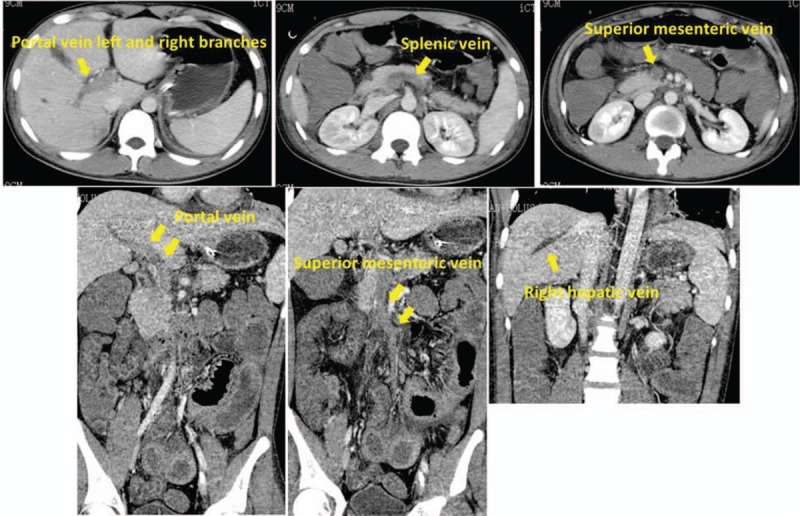
Whole abdominal enhanced computed tomography scan on July 25, 2017. Both axial and reconstructed images showed extensive venous filling defects, suggesting extensive venous thrombosis that was not significantly improved since July 21. The thrombosis was accompanied by partial small intestine expansion and intestinal wall edema.

**Figure 4:**
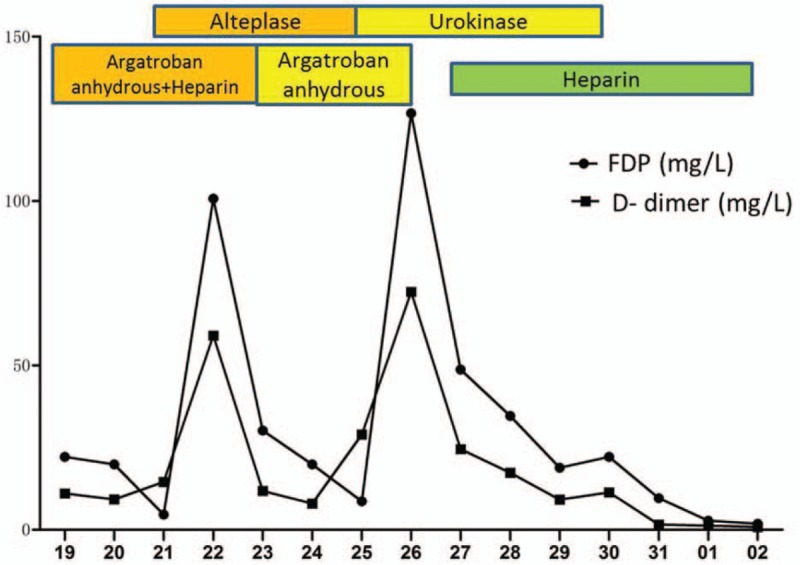
The variation trend of fibrinogen degradation products and D-dimer in our patient's plasma after anticoagulant and thrombolytic treatments. The coagulogram in this patient was monitored since his admission. The abscissa in the figure indicated different time points.

**Figure 5:**
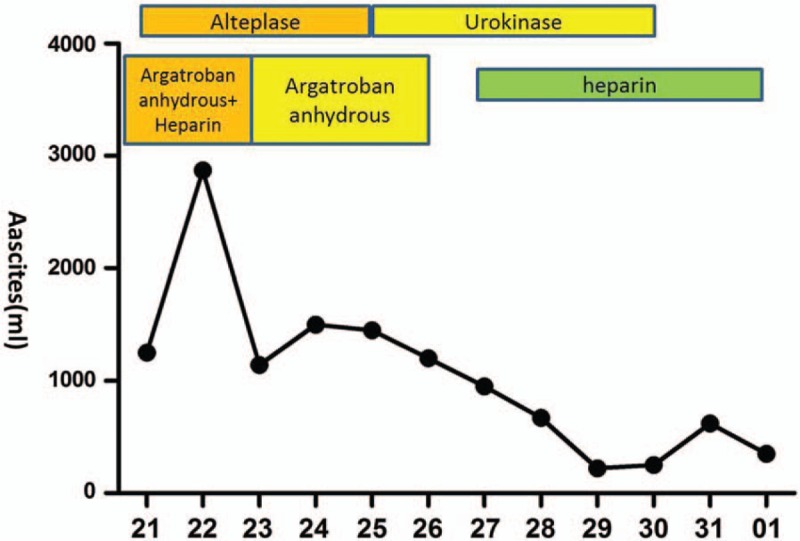
Changes in the volume of ascites of our patient. The abscissa in the figure indicated the daily ascites volumes.

**Figure 6:**
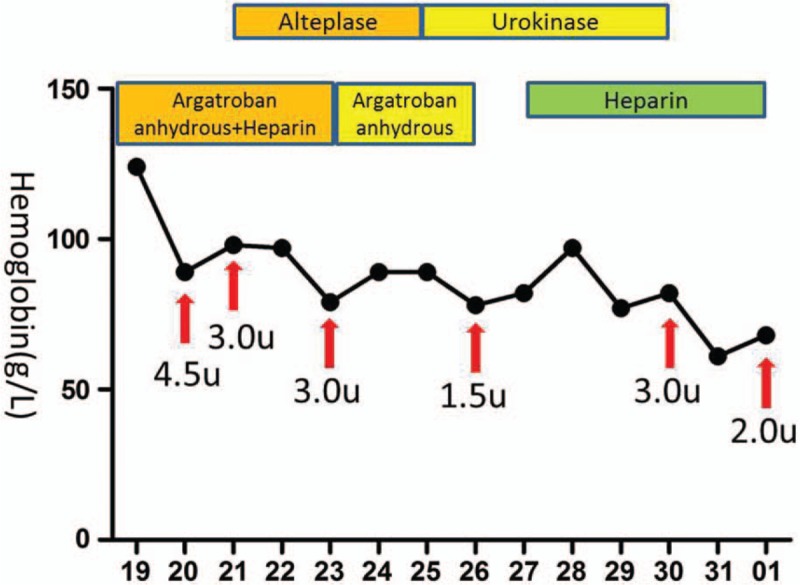
Changes in our patient's hemoglobin levels. The abscissa indicates the date when the hemoglobin level was measured, while the arrows show the amount of red blood cell transfusion on the indicated dates.

**Figure 7:**
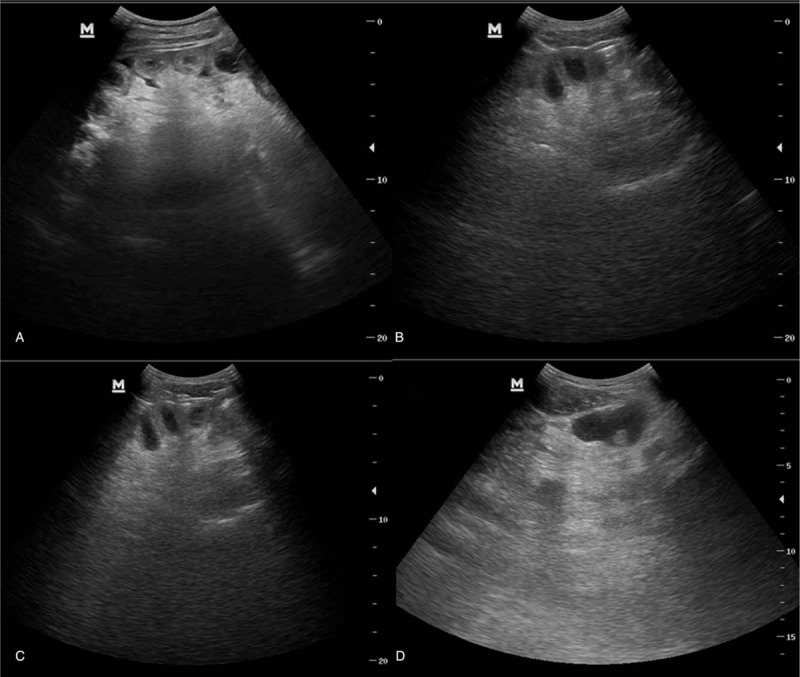
Bedside abdominal ultrasonography scan. (A) indicates intestinal cavity expansion and severe intestinal wall edema in this patient on July 21, 2017. (B) and (C) show that the intestinal wall still had significant edema on July 23, but the tension was reduced. (D) shows that the intestinal wall edema and tension were significantly reduced by July 25.

**Figure 8:**
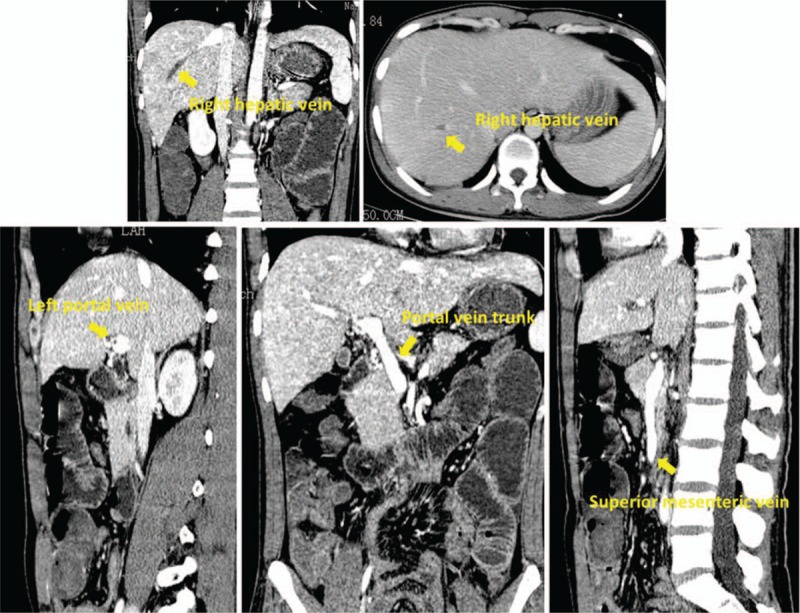
Whole abdomen enhanced computed tomography scan on July 31, 2017. Apart from the lack of significant improvement in the right hepatic vein thrombosis and small emboli in the left branch of the portal vein, the remaining veins were basically unobstructed. However, the small intestine showed expansion and effusion. A part of the small intestine was severely expanded with decreased perfusion, and intestinal necrosis could not be ruled out.

The patient's treatment regimens after admission were as follows:

(1) Methylprednisolone was intravenously injected at an initial dose of 40 mg q6 hours; however, after blood tests revealed a low eosinophil count, the methylprednisolone dose was gradually reduced to 20 mg/d.
(2) Plasma exchange and hemofiltration was performed. The plasma exchange treatment was performed once, where 3000 mL of plasma was replaced within 3 hours; continuous veno-venous hemofiltration was performed 4 times for a total of 85 hours with the blood flow volume set at 180 mL/min. The pre-dilution volume was 2000 mL, the post-dilution volume was 500 mL, and the ultrafiltration volume was 100 to 300 mL.
(3) Anticoagulant and thrombolytic treatments were administered; the patient received unfractionated heparin and argatroban as anticoagulants as well as alteplase and urokinase as thrombolytic agents. Both anticoagulant and thrombolytic drugs were continuously pumped through the deep vein. From July 19 to July 23, the patient was administered unfractionated heparin (200–1000 U/h) combined with argatroban (0.2–0.8 mg/h). However, after his platelet counts decreased, argatroban at the same dose was administered alone from July 24 to July 26 and was discontinued from July 27 to August 2, during which unfractionated heparin (maintained at 200–1000 U/h) was continuously administered as an anticoagulant. Alteplase (50–100 mg/d) was used for thrombolysis from July 21 to July 25. After 5 days of treatment, the patient's vital signs stabilized although he still had nausea, vomiting, bloating, abdominal pain, and intermittent bloody stool. Both axial and reconstructed images from a whole abdominal enhanced CT scan performed on July 25 showed extensive venous filling defects that suggested extensive venous thrombosis. This thrombosis had not significantly improved since July 21, and was accompanied by partial small intestine dilatation, intestinal wall edema, and decreased renal perfusion; moreover, renal artery thrombosis was not ruled out (Fig. 3). Alteplase was discontinued and replaced by urokinase (the initial dose was 200,000 U as a bolus injection, after which 50,000–100,000 U/h for 24 h was continuously pumped for maintenance) combined with unfractionated heparin (the dose of which [200–1000 U/h] was adjusted to maintain an activated partial thromboplastin time of 60–80 s) for enhanced anticoagulation. The levels of fibrinogen degradation products and D-dimer were continuously monitored during treatment (Fig. 4) to evaluate the thrombolytic effect. Starting on July 26, the patients symptoms, including vomiting and abdominal distension, began to improve, and his ascites gradually became lighter in color and decreased in volume (Fig. 5). At the same time, his bloody stool significantly abated, and his hemoglobin levels did not decrease significantly after intermittent blood transfusion (Fig. 6). As the patient had severe intestinal edema, small intestinal clearance, and a high risk for abdominal puncture at the time of his admission, abdominal puncture, and drainage were performed once his condition started to improve to reduce intraperitoneal pressure and enhance renal perfusion. After receiving anticoagulant and thrombolytic treatments, the patient's portal venous pressure was reduced, and his intestinal edema and exudate gradually decreased. Bedside abdominal ultrasonography indicated intestinal canal expansion and decreased edema (see Fig. 7). Moreover, an enhanced abdominal CT scan on July 31 showed that, while there remained no significant improvement in the right hepatic vein thrombosis and small emboli in the left branch of the portal vein, the remaining veins were essentially unobstructed and the small intestine was expanded and effused. A portion of the small intestine was severely expanded with decreased perfusion (Fig. 8); the urokinase was discontinued and heparin continuously administrated at 200–1000 U/h for 24 h) for anticoagulation.
(4) Meanwhile, the patient also received a blood transfusion, fluid infusion, and vasoactive drugs to maintain blood pressure; the patient also received anti-infection agents, somatostatin, and hypophysin to reduce portal pressure as well as symptomatic and supportive treatments. On August 12, the patient was found to have dark red bloody stools, decreased hemoglobin, right mid-abdominal pain, and board-like rigidity of the abdomen suggesting intestinal necrosis. The patient underwent emergency partial small intestine resection, following which his adverse abdominal symptoms improved. Enhanced whole abdominal CT on August 21 revealed that the right hepatic vein had less thrombosis than before and was completely unobstructed; meanwhile, the intestinal expansion and effusion were relieved. The patient was discharged on September 1, 2017. He was prescribed warfarin 2.5 mg/d orally after discharge. His prothrombin time/international normalized ratio was maintained between 1.5 and 2.5, and prednisone 5 mg/d was administered. During regular follow-up, this patient reported no discomfort, and his eosinophil levels were normal.

The study was approved by the Nantong Third People's Hospital Ethics Committee. The patient provided written informed consent for the publication of his details.

## 3 Discussion

HES is a rare clinical syndrome characterized by increased eosinophils in the bone marrow, peripheral blood, tissues, and organs, as well as damage to these tissues and organs caused by eosinophil infiltration. While the incidence of HES is low, its morbidity rate is high in patients with concurrent severe complications. A retrospective study at the Mayo Clinic found that 23 of 247 patients diagnosed with HES died, indicating a mortality rate of 9.3%.^[3]^

In 1975, Chusid et al^[5]^ first proposed the following criteria for the diagnosis of HES: peripheral blood eosinophils increase to above 1.5 × 10^9^/L for a period of at least 6 months; parasitic infections, drug allergies, and allergic diseases are ruled out as the causes of eosinophilia; and corresponding organ infiltration and injury are evident. The eosinophil count in our patient continuously increased to reach 7.47 × 10^9^/L, and infiltration injury occurred in the lungs and portal system. Although temporary eosinophilia can be ruled out if it persists for 6 months, many investigators suggest that patients meeting 2 of the above 3 criteria can be diagnosed with HES; If eosinophilia that lasts for 6 months is a necessary diagnostic criterion, the diagnosis and treatment of patients may be delayed.^[6]^ Moreover, there are controversies over the definition of organ injury caused by eosinophil infiltration. While histopathological findings of eosinophil infiltration and organ dysfunction would ideally confirm the diagnosis, indirect examinations such as imaging tests for detecting thrombosis may be more amenable.^[7]^ Our patient experienced coughing and expectoration during the onset period that was accompanied by significantly increased eosinophil counts. When he was admitted to our department, his main manifestations were extensive thrombosis in the portal vein system and gastrointestinal bleeding, while the coughing and expectoration had significantly improved. As the patient had previously been healthy, the increased eosinophil counts with extensive deep venous thrombosis in the abdomen, which definitely existed during the onset period, were considered to be caused by eosinophil infiltration of the vascular endothelia.

Another important aspect is the etiology of eosinophilia, as the factors leading to this condition include^[8]^: allergic diseases, which usually lead to a slight increase in eosinophils, and include allergic rhinitis, asthma, and drug allergies; parasitic infections, which often lead to mild-to-moderate eosinophilia and commonly include flagellates, trichinella, roundworms, and hookworms; autoimmune diseases, where eosinophils are significantly increased as part of the body's inflammatory response; and conditions such as acute and chronic eosinophilic leukemia, some malignant tumors, and idiopathic eosinophilia. Our patient was previously healthy, had no history of allergies (including food and drug allergies), did not keep pets, and had no parasites or eggs in his stool. Post-admission examination showed that immune indicators and tumor markers were both negative, and bone marrow cytology indicated no leukemia. After hormonal treatment, the eosinophil count in the peripheral blood promptly fell to normal levels, and prednisone 5 mg/d was used as maintenance treatment to preserve his eosinophil counts at these normal levels; thus, he was provisionally diagnosed with idiopathic HES.

Organ injuries associated with HES arise from particles released by eosinophils, including cationic proteins and eosinophil-derived neurotoxins. However, the eosinophil counts in the peripheral blood do not always reflect the degree of invasion and injury of the tissue. Regardless, the primary goal of treatment remains the reduction of eosinophil counts in the blood and tissues.^[2]^

Glucocorticoids are a first-line treatment for idiopathic and *FIP1L1-PDGFRA* fusion gene-negative HES, while imatinib is the preferred treatment for *FIP1L1-PDGFRA*-positive HES.^[9]^ Second-line treatments for idiopathic HES include hydroxyurea, interferon-α, imatinib, and mepolizumab.^[10]^ Hormonal therapy serves to interfere with the transcription of proinflammatory cytokines that are essential for the maturation, proliferation, migration, and chemical induction of eosinophils. The median initial dose of prednisone is 1 mg/kg/d, although a higher dose should be used in critically ill patients. When the eosinophil count drops to a normal level and the clinical symptoms are improved, prednisone may be reduced to 10 mg/d. The duration of hormone treatment varies greatly from 2 months to 20 years. According to a retrospective study of HES treatment, the treatment response in 85% of patients was good.^[4]^ Our patient was critically ill and had severe abdominal complications at the time he was admitted to our department. Therefore, he received a higher glucocorticoid dose and simultaneously underwent plasma exchange to eliminate inflammatory cytokines and thereby inhibit the activation of eosinophils. His peripheral blood eosinophil count rapidly decreased to a normal level after treatment, and no new infiltration-related injuries occurred in the tissues and organs.

HES patients with more extensive organ infiltration have a worse prognosis, and the number of affected organs is an independent risk factor for mortality. Up to 25% of HES patients have thrombotic complications; such thromboses can occur in both arteries and veins, with venous thromboses being the most common. The occurrence of thrombosis also suggests a poor prognosis and necessitates immediate treatment.^[3,11]^

The infiltration of eosinophils into blood vessels leads to an inflammatory response in the vascular walls and subsequently to thrombosis, ultimately resulting in vascular occlusion.^[12]^ The mechanism by which eosinophilic diseases cause thrombosis remains unclear. Studies have found that cytokines, cytokine receptors, and chemokines, especially interleukin (IL)-5, IL-3, and granulocyte-macrophage colony-stimulating factor, play important roles in the activation, transport, survival, and degranulation of eosinophils. Activated eosinophils lead to injuries in the tissues and vascular endothelial cells. Certain cytotoxic cationic proteins in eosinophil granules (e.g., eosinophil peroxidase and eosinophil-mediated neurotoxic substances) can act as platelet agonists to increase vascular permeability, stimulate the activation of factor XII, and decrease the anticoagulant effect of heparin, thus promoting the formation of a thrombus.^[13]^

Thrombosis is the most serious complication of HES, and there are no guidelines for the prophylaxis and treatment of HES patients complicated with thrombosis. There is also some controversy regarding whether or not HES patients should receive preventative anticoagulant therapy. However, anticoagulant treatment is necessary for evidential events such as intracardiac thrombosis, deep venous thrombosis, or circulation embolisms. Our patient had severe thrombosis in his portal venous system, renal artery, and mesenteric artery, which led to severe acute gastrointestinal bleeding due to portal hypertension and intestinal necrosis during the latter period of his illness.

There is no research on the most optimal anticoagulants to administer to patients with thrombosis caused by HES. Commonly used anticoagulants include warfarin, heparin, or low molecular weight heparin, and the standard treatment for deep venous thrombosis is the sequential use of low molecular weight heparin and vitamin K inhibitors. Our patient also had reduced platelets; therefore, we selectively used heparin and argatroban as anticoagulants. Extensive portal vein thrombosis with acute portal hypertension leads to gastrointestinal bleeding and bloody ascites; therefore, it is essential to restore the portal vein blood flow. Because the portal vein is inaccessible to interventional thrombolysis, anticoagulation and low-dose thrombolysis are generally used to treat venous thrombosis. However, adoption of these measures in our patient did not improve his condition. After weighing its pros and cons, the regimen used to treat deep venous thrombosis was administered simultaneously. Of note, even though the time interval for intravenous thrombolysis was longer, the thrombolytic effect was decreased owing to the prolonged thrombolytic time; hence, high-dose urokinase and heparin could additionally be administered. Meanwhile, the effects of high-dose anticoagulants and thrombolytic treatments on hemoglobin, platelets, and coagulogram results were monitored to avoid bleeding. After the use of anticoagulant and thrombolytic drugs, the fibrinogen degradation product and D-dimer levels in our patient significantly increased, and an abdominal CT reexamination showed a smaller thrombus while bedside B ultrasonography indicated that the intestinal wall edema was subsiding. Moreover, symptoms such as gastrointestinal bleeding and abdominal distension were significantly improved, indicating that anticoagulant and thrombolytic treatments are effective and safe.

In summary, we reported a critically ill patient with HES in whom the use of unfractionated heparin and argatroban for anticoagulation and of alteplase for thrombolysis produced poor results. However, the patient was ultimately cured following thrombolytic treatment with a larger dose of urokinase combined with heparin anticoagulation. This was achieved even though the patient had extensive thrombosis in the portal venous system and renal arteries caused by idiopathic HES, with concurrent gastrointestinal bleeding and intestinal necrosis. Hence, anticoagulant and thrombolytic treatments appear to be critical for treating patients with severe HES. As long as strict monitoring is performed, this treatment regimen also appears to be safe. Our report may be used as a reference for the diagnosis and treatment of this rare disease.

## Author contributions

**Conceptualization:** Xudong Han.

**Data curation:** Jinfeng Lin.

**Investigation:** Jinfeng Lin, Xiaoying Huang, Weihua Zhou, Suyan Zhang, Weiwei Sun, Yadong Wang, Ke Ren, Lijun Tian, Junxian Xu, Zhilong Cao, Zunguo Pu, Xudong Han.

**Resources:** Xudong Han.

**Writing – original draft:** Jinfeng Lin.

**Writing – review & editing:** Jinfeng Lin.
